# Chelating Ligand-Mediated Hydrothermal Synthesis of Samarium Orthovanadate with Decavanadate as Vanadium Source

**DOI:** 10.1155/2013/127816

**Published:** 2013-08-28

**Authors:** Quanguo Li, Wenli Zuo, Feng Li

**Affiliations:** College of Chemistry, Key Lab of Environment Friendly Chemistry and Application in Ministry of Education, Xiangtan University, Xiangtan 411105, China

## Abstract

A new ethylenediaminetetraacetic acid- (EDTA-) mediated hydrothermal route to prepare chrysanthemum-shaped samarium orthovanadate (SmVO_4_) nanocrystals with decavanadate (K_6_V_10_O_28_ *·*9H_2_O) as vanadium source has been developed. The present hydrothermal approach is simple and reproducible and employs a relatively mild reaction temperature. The EDTA, pH value, and temperature of the reaction systems play important roles in determining the morphologies and growth process of the SmVO_4_ products. The products have been characterized by X-ray diffraction (XRD), scanning electron microscopy (SEM), Fourier transform infrared spectroscopy (FT-IR), photoluminescence spectra (PL), and UV-Vis spectroscopy.

## 1. Introduction

Controllable synthesis of well-defined nanocrystals with uniform size and morphology such as wires, belts, tubes, cubes, and rods has attracted a worldwide interest due to their unique electronic, optical, and magnetic properties associated with the reduced dimensionality and their potential applications in nanotechnology [1–5]. As a consequence, various nanocrystals such as elemental semiconductors, II–VI and III–V semiconductors, metal oxides, metals, and inorganic salts on large scale have been prepared through all sorts of methods including templating direction, catalytic growth, electrochemistry, chemical vapor deposition, and solution-based solvothermal or hydrothermal treatment [6–13]. In the synthesis of nanocrystals, there are two challenges now: (1) the morphology is restricted by the nature of crystals, which makes it difficult to control the morphology of the product; (2) the products always deviate from our research target. In recent reports, a special “capping reagent” has been used in the synthesis of nanocrystals. The “capping reagent” is used for tailoring the crystal growth and may also provide the possibility of breaking the nature of crystals, which may lead to more different morphologies and wider applications [14–16].

In recent years, SmVO_4_ nanocrystals have been widely studied as a prospective material because they exhibit unique properties and applications in many fields, such as catalysis [17], gas sensors [18], optical polarizers [19], lithium intercalated electrodes [20], thin film phosphors [21], laser host materials [22], solar cells [23], and unusual magnetic materials [24]. For example, SmVO_4_ nanocrystals exhibit high activity and selectivity as catalysts for many organic reactions, such as oxidative dehydrogenation of alkanes, olefins, ethylbenzene, and oxidation of hydrogen sulfide to elemental sulfur [25–35].

To date, most of the syntheses of SmVO_4_ nanocrystals adopt solid-state reaction and citrate method [36], which needs harsh conditions. For example, Tojo et al. synthesized SmVO_4_ crystals by milling the mixture of single oxides of Sm_2_O_3_ and V_2_O_5_ powders at 1000–1100°C [37]. Thus, it is expected to develop a low cost and convenient method for the synthesis of SmVO_4_ nanocrystals with well-defined sizes and shapes [38]. For this purpose, the hydrothermal method has been considered as one of the most convenient methods for the controlled synthesis of SmVO_4_ nanocrystals [39]. In the preparation of SmVO_4_, most syntheses adopted VO_3_^−^ as the vanadium source, so it may be a good strategy for preparing SmVO_4_ with a new vanadium precursor [40]. Isopolyanions (e.g., decavanadate) may be a good candidate. In aqueous solutions, isopolyanions have versatile structures, and the structure of isopolyanions can be controlled by controlling the pH value. It means that we can obtain the target structure by adjusting the pH to a certain value, which is important for the preparation of aimed productions in the system of Sm-V-O. To the best of our knowledge, there are no reports on the synthesis of SmVO_4_ with novel precursor decavanadate by the hydrothermal method yet.

In this paper, we synthesized chrysanthemum-shaped SmVO_4_ nanorods from the reaction of K_6_V_10_O_28_ *·*9H_2_O and SmCl_3_ *·*XH_2_O precursors in the presence of capping reagent (EDTA) by hydrothermal method. The effects of the capping reagent, solution pH value, and reaction temperature on the shapes of the obtained SmVO_4_ nanocrystals have been studied. The possible mechanism for the formation of SmVO_4_ nanocrystals with various shapes is also proposed. 

## 2. Experimental Section

### 2.1. Preparation of Decavanadate

The decavanadate (K_6_V_10_O_28_ *·*9H_2_O) was prepared according to a described procedure [41].

### 2.2. Preparation of SmVO_4_ Products

SmVO_4_ products were prepared by hydrothermal method in the presence of capping reagent (EDTA). All reagents were analytical grade pure and used without further purification. In a typical procedure, 0.107 g K_6_V_10_O_28_ *·*9H_2_O and 0.215 g ethylenediaminetetraacetic acid (EDTA) were dissolved into 30 mL distilled water, respectively. Stirring for 30 min, 0.073 g SmCl_3_ *·*XH_2_O was dissolved into 20 mL distilled water. SmCl_3_·XH_2_O solution was added into K_6_V_10_O_28_·9H_2_O solution. After stirring for 10 min, EDTA solution was added into the solution, and 20 min later, sodium hydroxide solution (1 mol/L) was added dropwise into the solution to adjust the pH to the designed range (from 4 to 10). The resulting suspension was transferred into a 100 mL Teflon-lined stainless steel autoclave and sealed tightly. Hydrothermal synthesis was carried out at 140~180°C for 48 h in an electric oven without shaking or stirring. After cooling to room temperature naturally, the precipitates were collected, washed with distilled water and absolute ethanol for several times, and then dried in a vacuum oven at about 60°C for 10 h.

### 2.3. Characterization of SmVO_4_ Products

The pH values of the solution were measured by Leici pH Meter of Shanghai Precision and Scientific Instrument Corporation China. The phase purity and crystal structure of the obtained products were examined by X-ray diffraction (XRD) using a MiniFlex2 goniometer, employing a scanning rate of 0.02° s^−1^ in the 2*θ* range of 5°–70°. The operation voltage and current were maintained at 30 kV and 15 mA, respectively. The size distribution and morphology of the products were analyzed by scanning electron microscopy (SEM) (JSM-6610LV). The UV-Vis spectra of the as-synthesized SmVO_4_ nanocrystals were recorded on a Lambda 25 spectrometer. Fourier transform infrared spectroscopic (FTIR) analysis was carried out in the region of 4000-400 cm^−1^ by a PerkineElmer spectrometer instrument (pressed KBr disks). Room temperature photoluminescence (PL) spectra were recorded on a Hitachi 850 fluorescence spectrometer with Xe lamp as the excitation source at 25°C.

## 3. Results and Discussion

### 3.1. Structural Analysis

**Figure 1:**
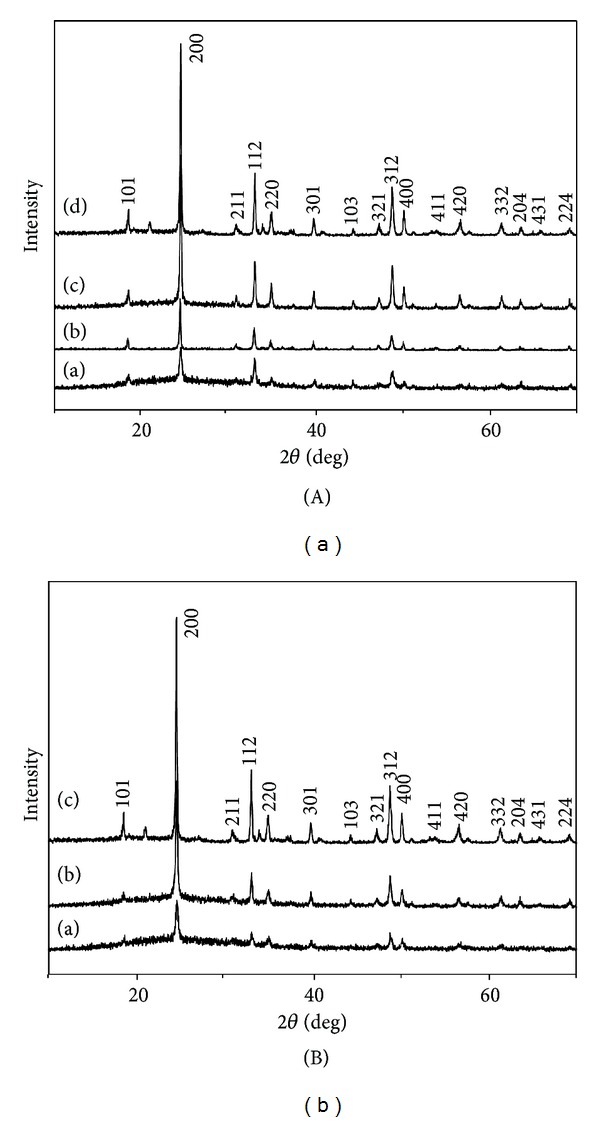
X-ray diffraction patterns of SmVO_4_ nanocrystals obtained (A) under 180°C, with different pH values: (a) EDTA, pH = 4; (b) EDTA, pH = 6; (c) EDTA, pH = 8; (d) EDTA, pH = 10, and (B) under pH = 10, with different temperatures: (a) EDTA, 140°C; (b) EDTA, 160°C; (c) EDTA, 180°C.

The phase identification of all the products was performed by XRD.  Figure 1(a) shows the XRD patterns of the products obtained via hydrothermal method with deferent pH values. The products synthesized with pH values of 4, 6, 8, and 10 show similar patterns, which can be indexed as the tetragonal zircon phase of SmVO_4_ with lattice contents of *a* = *b* = 7.265 Å and *c* = 6.389 Å, and are in good agreement with the standard values for the tetragonal phase SmVO_4_ (*Fm3hm*, JCPDS no. 17-0876) [42]. No XRD peaks correspond to single metal oxides of V_2_O_5_ [43], and Sm_2_O_3_ [44] were detected, indicating that the pure SmVO_4_ nanocrystals with tetragonal zircon-type structure can be obtained by this method. From Figure 1(a), we observed that the crystallinity of obtained products increased with the increasing of the pH value of the solution, when we kept the reaction temperature at 180°C. Obviously, the pH value of the solution plays an important role in the preparation of pure SmVO_4_ by hydrothermal method.

 To investigate the effect of temperature on the crystallinity, SmVO_4_ was prepared at different reaction temperatures. When the reaction temperature was 140°C, the crystallinity of the as-prepared product was low, and few peaks can be observed. Therefore, a higher temperature may help the formation of the thermodynamically stable, well-crystallized, and uniform products. It is very clear that the crystallinity of the obtained products increases with the increasing of the reaction temperature at pH = 10 (Figure 1(b)). Thus, we conclude that the solution pH value and temperature of the reaction systems play important roles in determining the crystallinity of the SmVO_4_ nanocrystals. Moreover, it can also be concluded that the formation of the pure tetragonal SmVO_4_ phase results in complete reaction of the 1 : 1 ratio of Sm^3+^ cation and VO_4_^3−^ anion monomers in the bulk solution during the synthesis. 

**Figure 2:**
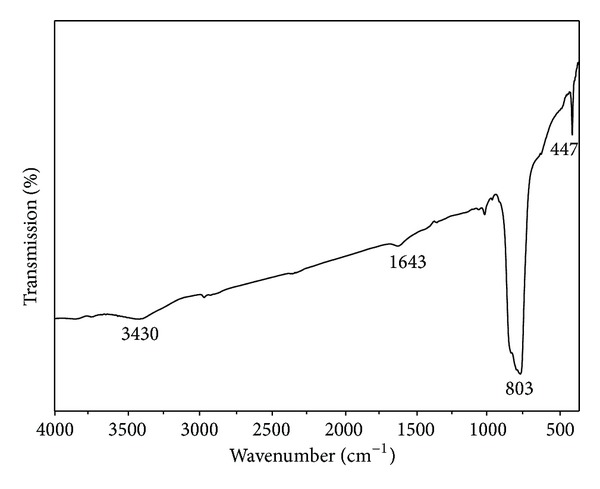
The FT-IR spectrum of SmVO_4_ nanocrystals synthesized under 180°C, EDTA, pH = 10.

 To further confirm the formation of the SmVO_4_, Fourier transform infrared (FT-IR) spectroscopy was performed (Figure 2). The weak band at 447 cm^−1^ is due to the *ν*_4_ antisymmetric deformation, and the strong absorption band at 803 cm^−1^ corresponds to the *ν*_3_ vibrational mode that belongs to antisymmetric stretching of VO_4_ tetrahedron [45]. It shows that the crystalline phase of SmVO_4_ formed in the as-prepared product. The absorption band located at 3430 cm^−1^ and 1643 cm^−1^ can be ascribed to the O–H stretching and bending vibrations of free water molecules adsorbed by products from the aqueous solution.

### 3.2. Morphology Control

**Figure 3:**
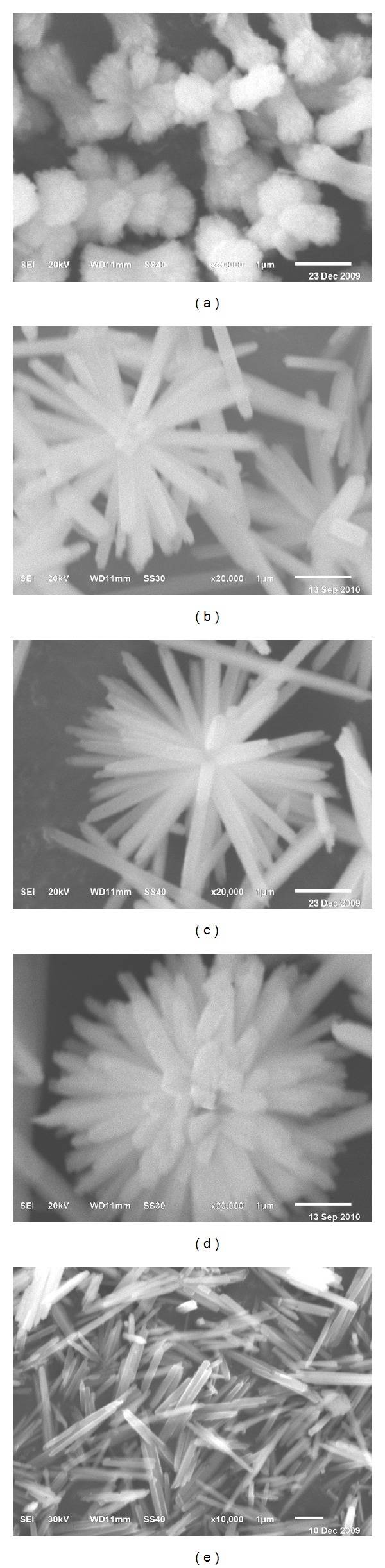
SEM images of SmVO_4_ nanocrystals synthesized under 180°C, with different reaction conditions: (a) EDTA, pH = 4; (b) EDTA, pH = 6; (c) EDTA, pH = 8; (d) EDTA, pH = 10; (e) absence of EDTA, pH = 6.

The morphology and structure of the as-prepared SmVO_4_ nanocrystals were further characterized by SEM.  Figure 3 shows the typical SEM images of the SmVO_4_ nanocrystals at the reaction temperature of 180°C. When the pH is lower than 4, the obtained products take on a dumbbell-shaped structure (Figure 3(a)). It is noted that the centers of several dumbbells are connected together. When the pH is higher than 8, the obtained products are chrysanthemum shaped with a high density of nanorods (Figure 3(d)). When the pH values are higher than 4 and lower than 8, the chrysanthemum-shaped structure appears with a lower ratio. As shown in Figure 3(b), we can observe that the amount of nanorods is relatively rare. With the increasing of the pH value, the density and aspect ratio of those nanorods are improved (Figures 3(c) and 3(d)). These results indicate that pH value is a key factor in the formation of the chrysanthemum-shaped structure of SmVO_4_ nanocrystals.

**Figure 4:**
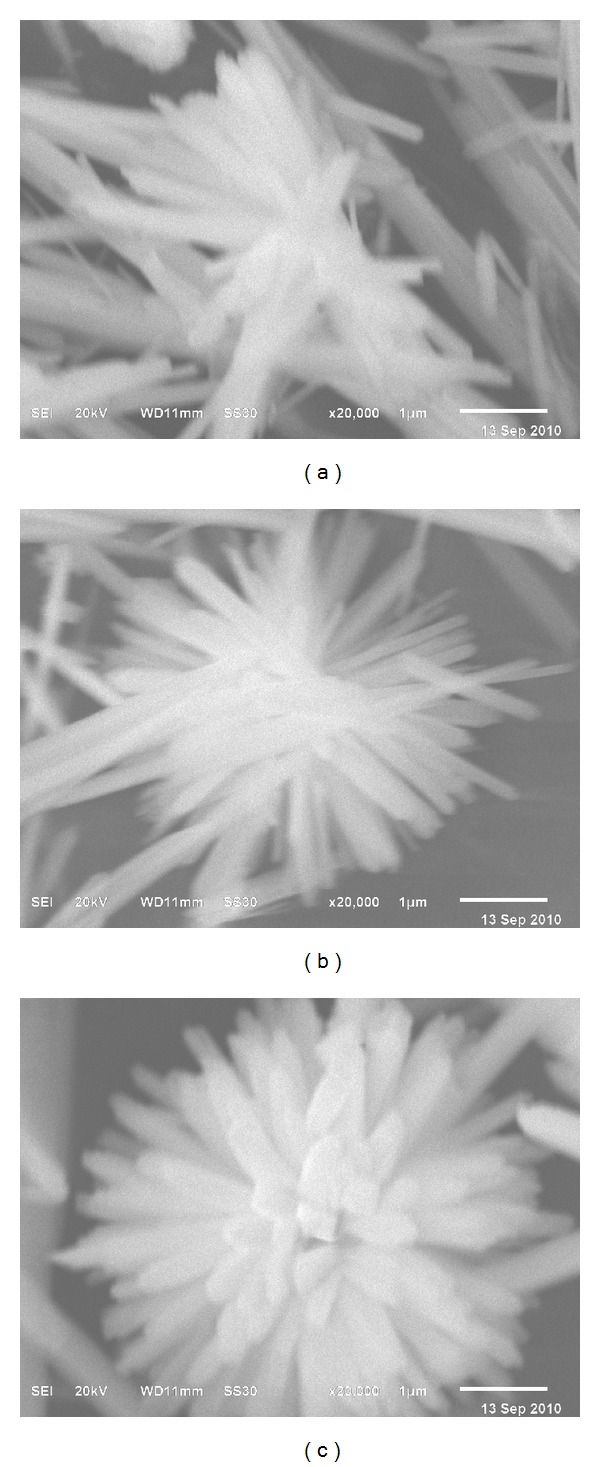
SEM images of the SmVO_4_ nanocrystals synthesized under pH = 9.7, with different reaction temperatures: (a) EDTA, 140°C; (b) EDTA, 160°C; (c) EDTA, 180°C.

In order to better understand the formation and evolution of SmVO_4_ nanostructures with the reaction temperature, reactions at 140°C and 160°C with the pH value of 10 were carried out. The SEM image of the product obtained from hydrothermal reactions at 140°C was shown in Figure 4(a). It is clearly shown that the as-synthesized products are composed of nanorods with inhomogeneous size distribution. When the reaction temperature was increased to 160°C, a large number of nanorods formed (Figure 4(b)). Some of them arranged regularly, and the density is higher than that in Figure 4(a). However, when the reaction temperature was increased to 180°C, the chrysanthemum-shaped structure of nanorods appeared, and the density and aspect ratio were further improved (Figure 4(c)). These results indicate that the reaction temperature has an important effect on the formation of chrysanthemum-shaped SmVO_4_ nanocrystals.

### 3.3. Formation Mechanism

Without EDTA in the reaction system, the tetragonal SmVO_4_ precipitates are aggregates of highly nanorods with inhomogeneous size distribution, and those nanorods were arranged inconstantly (Figure 3(e)). When EDTA was introduced into the reaction system, SmVO_4_ products with a high aspect ratio and density can be obtained, if the pH value and the reaction temperature are well controlled. These initial products can be mediated by the ligands adsorbed on the surface of crystals, and they will serve as seeds for the growth of highly anisotropic nanostructures in the solution-solid process via the dissolution and crystallization mechanisms [46, 47]. The reason of introducing EDTA is based on its chelating and capping effects, which will influence the growth rate of different facets distinguishingly. Once the EDTA is dissociated from the Sm^3+^ ions, it will bind to the specific surface of the precipitated SmVO_4_, which directly affects the facet growth and crystallinity of the nanocrystals [48]. The reason that the pH values influence the morphology of SmVO_4_ nanocrystals can be easily understood by taking the following considerations. When the pH value is lower than 4, V_10_O_28_^6−^ is stable, and the product is a dumbbell-shaped framework [49]. Since Sm^3+^ exists near the surrounding of anion framework, we suppose that the nucleation happens at the interface. When the framework is broken due to the increasing of the pH value of the reaction system, polyorthovanadate anion turns into VO_4_^3−^ ions and forms chrysanthemum-shaped structure. When pH is kept at 10, VO_4_^3−^ anion is the main form of orthovanadate, so no dumbbell shapes are observed. When the pH of the reaction system is kept at lower value, the aspect ratio of the chrysanthemum-shaped nanostructure of SmVO_4_ is reduced because of the weakened chelation and capping abilities of EDTA. Moreover, the heat treatment temperature also plays important roles in determining the shape of the products. The strong ligand (EDTA) plays two roles. It is not only required to form a stable complex with Sm^3+^ but also acts as a capping reagent binding to the surface of crystals, which directly affects the facet growth and crystallinity of the nanocrystals. Therefore, we can control the aspect ratio and the density of SmVO_4_ nanocrystals by adjusting the pH value and the reaction temperature when EDTA is introduced.

### 3.4. UV-Vis Spectroscopy and Photoluminescence Properties of SmVO_4_ Nanocrystals

**Figure 5:**
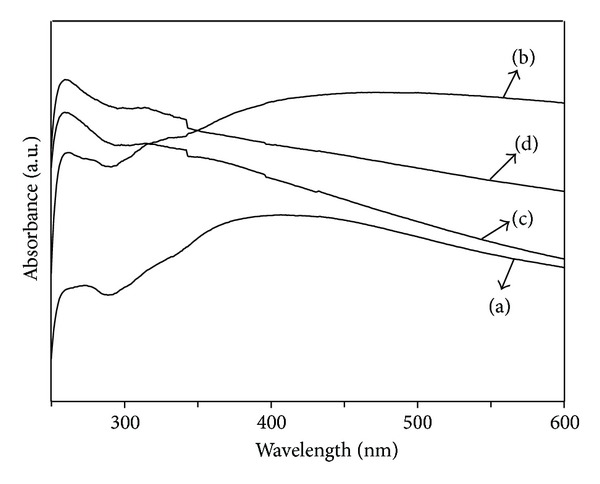
UV-Vis spectra of the as-synthesized SmVO_4_ nanocrystals under 180°C, with different pH values: (a) EDTA, pH = 4; (b) EDTA, pH = 6; (c) EDTA, pH = 8; (d) EDTA, pH = 10.

UV-Vis spectroscopy has been used for characterizing the optical property of the SmVO_4_ nanocrystals. Examples for the UV-Vis absorption spectra experiments were prepared by dispersing the as-prepared SmVO_4_ nanocrystals in ethanol with sonication bath for 30 min to form clear solutions.  Figure 5 gives the UV-Vis absorption spectra of the SmVO_4_ nanocrystals obtained at different pH values of 4, 6, 8, and 10. As shown in Figure 5, the absorption peaks of the products at about 250 nm are attributed to the charge transfer from the oxygen ligands to the central vanadium atom inside the VO_4_^3−^ groups in the samarium orthovanadate [50].

**Figure 6:**
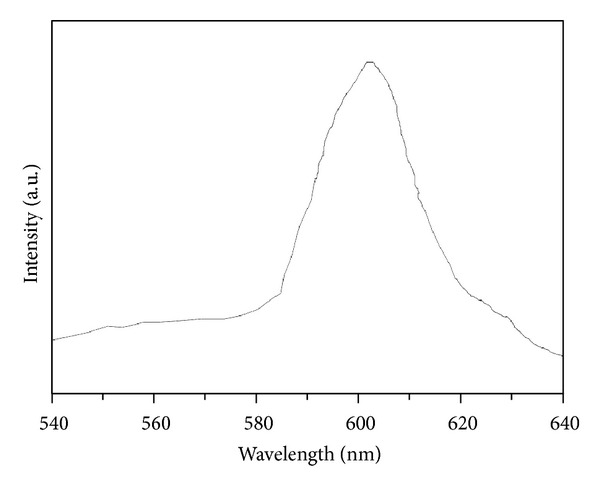
Room temperature photoluminescence spectra of tetragonal SmVO_4_ nanocrystals obtained after the hydrothermal treatment under 180°C, EDTA, pH = 10.

The room temperature photoluminescence spectrum of the as-synthesized SmVO_4_ with the excitation of 288 nm was shown in Figure 6. It shows one strong and broad red emission at 605 nm, which is a characteristic transition from ^4^G_5/2_ to ^6^H_7/2_ states of Sm (III) [50].

## 4. Conclusions

In summary, chrysanthemum-shaped SmVO_4_ nanorods have been successfully prepared with a novel precursor decavanadate in the presence of EDTA. It was found that the pH value, the reaction temperature, and the introduction of EDTA play key roles in the morphology evolution of the products. In addition, a possible growth mechanism of SmVO_4_ with the morphology of chrysanthemum was discussed. Due to the simplicity of this system and the efficient control over the morphology, we suppose that this method may have wide applications in exploring the crystal growth process and provide guidance for the morphology controllable synthesis of other functional inorganic materials.
